# Tumor aromatase expression as a prognostic factor for local control in young breast cancer patients after breast-conserving treatment

**DOI:** 10.1186/bcr2343

**Published:** 2009-07-28

**Authors:** Marc A Bollet, Alexia Savignoni, Leanne De Koning, Carine Tran-Perennou, Catherine Barbaroux, Armelle Degeorges, Brigitte Sigal-Zafrani, Geneviève Almouzni, Paul Cottu, Rémy Salmon, Nicolas Servant, Alain Fourquet, Patricia de Cremoux

**Affiliations:** 1 Department of Radiation Oncology, Institut Curie, 26 rue d'Ulm, 75248 Paris, France; 2 Department of Biostatistics, Institut Curie, 26 rue d'Ulm, 75248 Paris, France; 3 Laboratory of Nuclear Dynamics and Genome Plasticity (UMR 218), Institut Curie, 26 rue d'Ulm, 75248 Paris, France; 4 Department of Tumour Biology, Institut Curie, 26 rue d'Ulm, 75248 Paris, France; 5 Department of Medical Oncology, Institut Curie, 26 rue d'Ulm, 75248 Paris, France; 6 Department of Surgery, Institut Curie, 26 rue d'Ulm, 75248 Paris, France; 7 Department of Bio-informatics, Institut Curie, 26 rue d'Ulm, 75248 Paris, France; 8 INSERM, U900, 26 rue d'Ulm, 75248 Paris, France; 9 Ecole des Mines de Paris, 35 rue Saint Honoré, 77300, Fontainebleau, France

## Abstract

**Introduction:**

We sought to determine whether the levels of expression of 17 candidate genes were associated with locoregional control after breast-conserving treatments of early-stage breast cancers in young, premenopausal women.

**Methods:**

Gene expression was measured by using RT-PCR in the breast tumors of a series of 53 young (younger than 40 years), premenopausal patients. All treatments consisted of primary breast-conserving surgery followed by whole-breast radiotherapy (± regional lymph nodes) with or without systemic treatments (chemotherapy ± hormone therapy). The median follow-up was 10 years.

**Results:**

The 10-year locoregional control rate was 70% (95% CI, 57% to 87%). In univariate analysis, no clinical/pathologic prognostic factors were found to be significantly associated with decreased locoregional control. Expression of three genes was found to be significantly associated with an increased locoregional recurrence rate: low *estrogen-receptor β*, low *aromatase*, and high *GATA3*. Two others were associated with only a trend (*P *< 0.10): low *HER1 *and *SKP2*. In multivariate analysis, only the absence of aromatase was significantly associated with an increased locoregional recurrence rate (*P *= 0.003; relative risk = 0.49; 95% CI 0.29 to 0.82).

**Conclusions:**

Recent data give credit to the fact that breast cancer in young women is a distinct biologic entity driven by special oncogenic pathways. Our results highlight the role of estrogen-signaling pathways (mainly *CYP19/aromatase*, *GATA3*, and *ER-β*) in the risk of locoregional recurrence of breast cancer in young women. Confirmation in larger prospective studies is needed.

## Introduction

Breast-conserving therapy is the preferred treatment for patients with early-stage breast cancer [1]. It offers equal local control and overall survival [2], as well as superior psychosocial outcomes compared with modified radical mastectomy [3,4]. Locoregional recurrences can be traumatizing and even fatal, despite aggressive therapies [2]. Young age is generally considered to be the most important risk factor for locoregional recurrence after breast-conserving treatments [5-7]. This higher risk, which is not yet understood despite numerous studies, could find its explanation in tumor biology.

The hormonal environment, with menopause as its archetype epitome, is the major physiological difference between younger and older patients. Estrogens not only are the main regulators of growth and differentiation in the normal mammary gland, but also play a major role in the onset and progression of breast cancer [8,9] (reviewed by Yager [10]). Other signaling pathways, not directly related to estrogen receptors (ERs), also are involved in the growth of epithelial tissues.

In premenopausal breast cancer patients, little is known about the expression levels of genes that are directly or indirectly involved in hormone (especially ER) and growth factors signaling pathways. The aim of this study, conducted in a series of women diagnosed with invasive breast cancers before the age of 40, was therefore to determine the relation between locoregional relapse, classic biopathologic factors, and the intratumoral levels of gene expression of 17 hormone receptors, growth-factor receptors, or proliferation genes: *ERα*, *ERβ*, *progesterone receptor *(PR), *nuclear receptor co-repressor (NCoR), nuclear receptor coactivator 3 (NCoA3/AIB1), aromatase (CYP19), GATA-binding protein 3 (GATA3), human epidermal receptor (HER) 1 to 4, insulin-like growth factor 1-receptor (IGF1R), antigen identified by monoclonal antibody ki-67 (MKI67), cyclin E1 (CCNE1), cyclin E2 (CCNE1), S-phase kinase-associated protein 2 (SKP2)*, and the two subunits of *chromatin assembly factor 1 (CAF-1 p150 *and *CAF-1 p60)*. Quantitative reverse-transcriptase polymerase chain reaction (RT-PCR) was chosen, as it is the most precise method to measure absolute levels of expression of selected target genes within a wide range, from very high to very low transcript levels [11,12].

## Materials and methods

### 1.1 Patients and tissue specimens

Between 1988 and 1999, 257 premenopausal women, younger than 40 years, with no previous history of cancer were treated at the Institut Curie for early unilateral breast cancers. The present retrospective study was based on 53 of these 257 patients for whom frozen tumor tissue was available. Median age at diagnosis was 37 years (range, 23 to 40) with 30% of patients (16 patients) no older than 36 years. Median follow-up was 10 years (range, 2 to 18 years).

Patient and tumor characteristics are reported in Table 1. Clinical stage [13] was either T1 or T2, N0 or N1. All specimens were reviewed by the same pathologist (BSZ). Histologic classification of the infiltrating carcinomas was reported according to the World Health Organization criteria, and histologic grade was reported according to Ellis and Elston [14]. The median number of mitoses, calculated per 10 high-power fields [15], was 13 (2 to 120). Hormone receptors (HRs) were positive when nuclear staining for either estradiol receptors (ERs) or progesterone receptors (PRs) was observed in at least 10% of invasive cells with immunohistochemistry [16]. No pathologic axillary lymph node involvement was observed in 31 (58%) patients.

**Table 1:** Patient characteristics

	RT-PCR series		Whole series		
			
Total	*n*	%	*n*	%	*P*
Family history of breast cancer (MD = 0 and 1)^a^					0.77
Without	42	79	198	77	
With	11	21	58	23	
Age (MD = 0 and 0)^a^					
Median (min-max) in years	37 (23–40)		37 (23–40)		0.71
≤ 35 years old	37	70	176	68	0.85
> 35 years old	16	30	81	32	
Clinical T stage (MD = 0 and 0)^a^					0.0496
cT0–1	31	58	186	72	
cT2	22	42	71	28	
Clinical N stage (MD = 0 and 0)^a^					0.70
N0	52	98	246	96	
N1	1	2	11	4	
Type of invasive carcinoma (MD = 0 and 0)^a^					0.18^b^
Ductal	46	87	223	87	
Lobular	6	11	16	6	
Other	1	2	18	7	
Histologic grade (MD = 0 and 1)^a^					0.03^b^
1–2	14	26	110	43	
3	36	68	120	47	
Unclassifiable	3	6	26	10	
Estrogen receptor (ER) (MD = 6 and 37)^a^					0.59
ER^-^	10	21	55	25	
ER^+^	37	79	165	75	
Progesterone receptor (PR) (MD = 6 and 39)^a^					0.84
PR^-^	9	19	39	18	
PR^+^	38	81	179	82	
Hormone receptors (HRs) (MD = 6 and 37)^a^					0.87
HR^-^	7	15	35	16	
HR^+^	40	85	185	84	
Lymphovascular involvement (MD = 3 and 43)^a^					0.0004
Absent	33	66	188	88	
Present	17	34	26	12	
Histologic T stage (MD = 5 and 12)^a^					0.89
pT1	34	71	176	72	
pT2	14	29	69	28	
Histologic N stage (MD = 0 and 34)^a^					0.06
pN0	31	58	182	73	
pN1–3	22	42	41	27	
Surgical margins (MD = 4 and 10)^a^					0.10
Satisfactory (≥ 3 mm)	32	65	129	52	
Unsatisfactory	17	35	118	48	
Systemic therapy (MD = 0 and 0)^a^					0.0001
None	16	30	151	59	
Hormone therapy (HT) only	0	0	101	39	
Chemotherapy ± HT	37	70	5	2	
Total RT dose (MD = 0)^a^					
Median (min-max) in Gy	66 (50–75)		64 (0–80)		0.14
< 60 Gy	13	25	101	39	0.043
≥ 60 Gy	40	75	156	61	

Characteristics of the rt-PCR series (53 patients) in comparison to the whole population of consecutive patients with the same selection criteria treated over the same period (1988–1999) at the Institut Curie (257 patients). MD = missing data (both in the rt-PCR and in the whole series); total RT dose = total radiotherapy dose to the tumor bed (whole-breast radiotherapy dose + boost dose). ^a^0.37 when the comparison excluded histologic types other than ductal or lobular. ^b^0.05 when the comparison included all histologic grades.

This study was approved by the Institutional Review Board and Ethics committee. Patients were informed that their biologic samples could be used for research purposes and that they had the right to refuse if they so wished.

### 1.2 Treatments

Surgery consisted of breast-conserving procedures as first-line treatment in all cases. The quality of the surgical margins was classified as wide (more than 3 mm) in 32 (65%) patients, close (3 mm or less) in 11 (22%) patients, involved with ductal carcinoma *in situ *in 2 (4%) patients, involved with invasive carcinoma in 4 (8%) patients, and unknown in 4 patients. Axillary lymph node dissection was performed in all patients.

Patients received posttumorectomy radiotherapy with a median dose of 51 Gy (range, 45 to 54 Gy) to the breast. A boost to the tumor bed was performed for 40 (75%) of patients with a median dose of 16 Gy (range, 7 to 25 Gy). The median total dose to the tumor bed was 66 Gy (range, 50 to 75 Gy). Supraclavicular irradiation was performed in 27 (51%) of patients. Internal mammary irradiation was performed in 38 (72%) of patients. In the case of lymph node involvement, the internal mammary chain and the supraclavicular area were both irradiated. In the absence of lymph node involvement, irradiation of the internal mammary chain, with or without the supraclavicular area, was indicated for centrally located tumors with histopathologic features of aggressiveness. Axillary irradiation was added in the presence of extensive axillary involvement or in the absence of axillary lymph node dissection.

No protocol to boost all young patients with negative surgical margins was available at that time, and some patients reported in this series were accrued in the EORTC boost trial that randomized between boost and no boost from 1989 to 1996 [5,17,18]. In the case of positive surgical margins, a radiotherapy boost of generally 20 to 28 Gy was added to the whole-breast irradiation. For patients not participating in the EORTC randomized trial, a boost of 10 to 16 Gy was added in the case of aggressive histopathologic features (unsatisfactory margins, high histopathologic grade, high proliferation index, absence of hormone receptors).

The reasons for absence of repeated excision were not always specified, but when they were specified, the reasons were the patient's choice not to undergo a new surgical procedure that could have been mastectomy.

No systemic therapy was administered in 16 (30%) patients. Chemotherapy only was given to 23 (43%) patients, consisting of anthracycline-based combination chemotherapy in most cases (usually six cycles of 5-fluorouracil, doxorubicin, and cyclophosphamide). A combination of chemotherapy and hormone therapy (tamoxifen for 5 years) was administered to 14 (26%) patients.

### 1.3 RNA isolation, RT-PCR

Total RNA was extracted from frozen tumor samples and first-strand cDNA synthesis was performed, as previously described from 1 μg of total RNA by using Superscript II RT (Invitrogen, Carlsbad, CA, USA) [19]. *ERα, ERβ, PR, HER1 to HER4, MKi67, Cyclin E1 and E2, GATA3, IGF1-R, NCor, NCoA3, CYP19 (aromatase gene), SKP2 *and *CAF-1 p150*, and *CAF-1 p60 *transcripts were quantified by using real-time quantitative reverse transcription-PCR assays. The nucleotide and probe sequences and the conditions of PCR were described previously for *ERα, ERβ, PR, HER1 to HER4, MKi67, cyclin E1 *and *E2*, *NCor, NCoA3, CYP19 *[see Additional data file 1] [19,20]. *GATA3, IGF1R *were quantified by Assays-on-Demand from Applied Biosystems (Applied Biosystems, Inc., Foster City, CA). The nucleotide and probe sequences *SKP2 *and *CAF-1 *were chosen with the help of Primer express software (Applied Biosystems). *TBP *(*TATA box binding protein*) was used as the endogenous reference gene for immediate quantification of transcripts. Searches were conducted in dbEST, htgs, and nr data bases [21] to confirm the total gene specificity of nucleotide sequences. Primers were placed at the junction between two exons. All PCRs were performed in duplicate by using ABI Prism 7700 Sequence Detection System (Perkin Elmer Applied Biosystems) and the Core reagent kit (Eurogentec). A 5-μl diluted sample of cDNA (12.5 ng) was added to 20 μl of the PCR mix. The thermal-cycling conditions comprised an initial denaturation step at 95°C for 10 min, and 45 cycles at 95°C for 15 s and either 60°C or 65°C, depending on the target, for 1 min.

Results were expressed as N-fold differences in target gene expression relative to a reference gene defined as "N target" (arbitrary units) and was determined as follows:



where E is the efficiency of PCR measured by using the slope of the calibration curve, and Ct is the cycle threshold.

### 1.4 Statistical analysis

Survival rates, defined from the date of surgery to the occurrence of the event, were estimated by using the Kaplan-Meier estimate; groups were compared by using the log-rank test. Event-free patients were censored at the date of their last known contact or death. Local relapses were defined as the occurrence of breast carcinoma (either invasive or ductal carcinoma *in situ*) in the treated breast. Locoregional relapses were our primary end point and were defined as either a local relapse or a recurrence in the ipsilateral lymph node areas (axillary, internal mammary, supraclavicular). Contralateral breast cancers could be either ductal *in situ *or infiltrating carcinoma. Distant disease was defined as disease occurring elsewhere than in the contralateral breast or locoregional site. Disease-free was defined as the absence of locoregional or distant relapses or both. Survival rates and relative risks (RRs) are presented with their 95% confidence intervals (CIs). Annual risks were calculated and plotted. The RT-PCR series of 53 patients was compared with the whole series of all consecutive patients treated over the same period (1988 through 1999) at the Institut Curie (257 patients) after exclusion of the RT-PCR patients. All gene expressions were analyzed as continuous variables to avoid having to search for cut-off values. Fisher's Exact or χ^2 ^tests were used to compare percentages, as appropriate. Univariate analyses were performed to identify prognostic factors of outcome and to estimate crude relative risks (RRs). The influence of each factor, adjusted to the others, was assessed in a multivariate analysis by the Cox proportional hazards model [22]. A stepwise modeling algorithm was used, with a limit of significance of 0.10 for entering and 0.05 for removing risk factors. The limit of significance was 0.05. Analyses were performed by using R software, 2.5.0.

## Results

The comparison of our series of patients with unselected, consecutive patients is summarized in Table 1. The patients analyzed with RT-PCR had tumors of higher clinical stage, higher histologic grade, and more lymphovascular invasion, and more often received chemotherapy and a total dose to the tumor bed higher than 60 Gy. All other patient, tumor, and treatment characteristics were similar.

Fifteen locoregional recurrences occurred (13 only local, 2 local and regional). Five-year and 10-year locoregional control rates were 82% (95% CI, 72% to 93%) and 70% (95% CI, 57% to 87%), respectively. All local recurrences occurred in the same quadrant as the primary tumor. Treatment of the local recurrence included salvage mastectomy with or without systemic therapy in 11 (73%) patients. In three (6%) patients, contralateral breast cancer developed. In 15 patients, distant metastases developed. Five-year and 10-year distant disease-free survival rates were 82% (95% CI, 72% to 93%) and 69% (95% CI, 56% to 85%), respectively. Five-year and 10-year overall survival rates were 85% (95% CI, 76% to 95%) and 78% (95% CI, 65% to 92%), respectively. In no patient did a second non-breast cancer develop.

**Figure 1:**
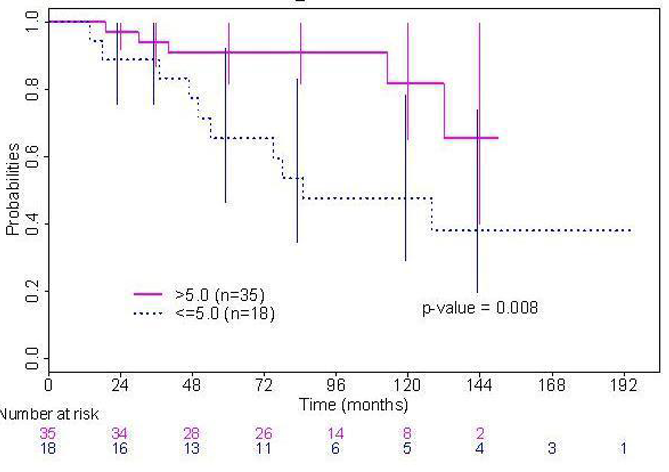
Locoregional-free recurrence interval according to the level of intratumoral expression of aromatase.

Because regional recurrence was always accompanied by local recurrence, risk factors for local and locoregional recurrence were the same (data not shown). In univariate analysis, no clinicopathologic prognostic factor was found to be significantly associated with decreased locoregional control (Table 2). In univariate analysis, three gene-expression levels were found to be significantly associated with an increased locoregional recurrence rate: low *ERβ*, low *aromatase*, high *GATA3 *(Table 3). Two other factors were associated only with a trend (*P *< 0.10): low *HER1 *and *SKP2*. Multivariate analysis was performed, taking the factors associated (*P *< 0.10) with locoregional recurrence into account in a Cox model (that is, 5 RT-PCR features (*ERβ, HER1, GATA3, SKP2 *and *aromatase*). Only low tumor expression of aromatase was significantly associated with an increased locoregional recurrence rate (*P *= 0.003; relative risk = 0.49; 95% CI, 0.29 to 0.82). Locoregional-free recurrence interval according to the level of intratumoral expression of aromatase with a cut-off at 5 is displayed in Figure 1. *GATA-3 *was associated with a trend toward an increased locoregional recurrence rate (*P *= 0.06; relative risk = 1.49; 95% CI, 0.02 to 2.39).

**Table 2:** Univariate analysis of clinical and histopathologic prognostic factors for locoregional control

	rt-PCR series			
	
	*n*	10-year LRC (%) [95% CI]	*P*^a^	RR [95% CI]
Family history of breast cancer			1.0	
No	42	70 [56–87]		1
Yes	11	NA		1 [0.22–4.59]
Age (continuous variable)			0.38	0.94 [0.83–1.07]
Age (years; dummy variable)			0.57	
> 35	16	73 [59–90]		1
≤ 35	37	60 [32–100]		1.37 [0.47–4.0]
Clinical tumor stage			0.71	
cT1	31	70 [53–91]		1
cT2	22	74 [56–96]		0.81 [0.28–2.39]
Histologic T stage			0.55	
pT1	34	70 [54–91]		1
pT2	14	70 [49–100]		0.71 [0.24–2.13]
Surgical margin			0.70	
≥ 3 mm	32	61 [43–86]		1
< 3 mm	17	81 [64–100]		0.81 [0.27–2.37]
Lymphovascular invasion			0.78	
Absent	33	71 [56–91]		1
Present	17	71 [50–100]		1.18 [0.36–3.87]
Histologic N stage			0.54	
pN0	31	63 [47–84]		1
pN1	16	78 [53–100]		0.49 [0.13–1.77]
pN2	6	80 [52–100]		0.77 [0.17–3.52]
Histologic type			0.14	
Ductal	46	75 [63–90]		1
Lobular	6	33 [7–100]		2.61 [0.72–9.53]
Estrogen receptors (ERs)			0.14	
ER^-^	10	90 [73–100]		1
ER^+^	37	63 [47–85]		4.28 [0.55–33.27]
Progesterone receptors (PR)			0.84	
PR^-^	9	71 [43–100]		1
PR^+^	38	69 [53–88]		1.18 [0.26–5.27]
Hormone receptors (HRs)			0.48	
ER^- ^and PR^-^	7	86 [63–100]		1
ER^+ ^or PR^+^	40	67 [52–86]		2.1 [0.06–3.72]
Histopathologic index			0.59	
Grade 1–2	14	64 [35–100]		1
Grade 3	36	73 [59–91]		0.70 [0.39–5.19]
Systemic therapy			0.35	
None	16	54 [33–86]		1
ChT	23	85 [70–100]		0.47 [0.15–1.49]
ChT + HT	14	43 [11–100]		0.48 [0.12–1.90]
Total RT dose^b^			0.27	
≥ 60 Gy	43	76 [59–97]		1
< 60 Gy	13	50 [28–88]		1.8 [0.63–5.15]

NA = not applicable; CI = confidence interval; RR = relative risk for locoregional recurrence. ^a^*P *values pertain to the relative risks. ^b^Total dose of radiotherapy to the tumor bed (whole breast dose + boost dose).

**Table 3:** Univariate and multivariate analyses of gene-expression prognostic factors (as continuous variables) for locoregional recurrences

Gene expression by quantitative rt-PCR (continuous variables)	Univariate analysis		Multivariate analysis	
		
	HR (RR) [95% CI]	*P*	HR (RR) [95% CI]	*P*
Estrogen receptor-α	1.22 [0.90–1.66]	0.15		
Estrogen receptor-β	0.61 [0.39–0.95]	0.04		0.54
Progesterone receptor	1.13 [0.88–1.45]	0.32		
HER1	0.65 [0.37–1.13]	0.10		0.72
HER2	0.89 [0.64–1.25]	0.49		
HER3	1.06 [0.55–2.02]	0.87		
HER4	1.22 [0.91–1.63]	0.14		
MKI 67	0.89 [0.61–1.30]	0.56		
Cyclin E1	0.81 [0.48–1.36]	0.43		
Cyclin E2	0.75 [0.49–1.14]	0.18		
GATA 3	1.61 [0.95–2.73]	0.04	1.49 [0.92–2.39]	0.06
IGF1R	1.41 [0.93–2.14]	0.13		
NCoR	0.90 [0.38–2.15]	0.82		
NCoA3/AIB1	0.51 [0.21–1.22]	0.14		
CYP19 (aromatase)	0.48 [0.28–0.80]	0.003	0.49 [0.29–0.82]	0.003
Skp2	0.52 [0.23–1.17]	0.10		0.18
CAF-1 p150	1.00 [0.48–2.12]	1.00		
CAF-1 p60	1.00 [0.97–1.03]	0.94		

RR = relative risk for locoregional recurrence calculated with the nonparametric Cox model; CI = confidence interval; HER = human epidermal receptor. Because of lack of prognostic values of the clinical variables in univariate analysis, none was entered in the multivariate analysis.

Associations between gene expressions and locoregional recurrences were looked for in two publicly available microarray studies by Kreike and colleagues [23] and Nuyten and associates [24] that gave information regarding the age of the young women (48 and 35 patients with 18 and 7 local recurrences, respectively) treated with breast-conserving treatments with whole-breast radiotherapy. They both failed to show an association between the gene expression of either aromatase or gata3 and local recurrences [see Additional data file 2].

## Discussion

This study was based on a series of 53 premenopausal women younger than 40 years with a long (10 years) follow-up treated at the Institut Curie for invasive breast cancer with primary breast-conserving surgery. Given the specificity of the hormonal environment in young patients, genes directly or indirectly involved in hormone and growth-factor signaling pathways were analyzed. In addition to the usual clinical and histopathologic features, the levels of expression of 17 candidate genes for an association with locoregional control also were examined.

To verify that these findings could be extrapolated, we compared the characteristics of patients and tumors of the 53 patients included in this study with the 257 other patients treated over the same period (1988 through 1999) at the Institut Curie who met the same inclusion criteria (age, medical history, therapeutic sequence) (Table 1). This comparison showed that this RT-PCR series consisted of patients with tumors with a more advanced clinical stage, higher histologic grade, and more lymphovascular invasion and who therefore more frequently received chemotherapy and a total dose to the tumor bed higher than 60 Gy. All other patient, tumor, and treatment characteristics were comparable. It could be hypothesized that these differences were due, at the time of this study (1988 to 1999), to technical parameters: tissue samples for frozen storage would be easier to obtain from larger tumors.

In this series of 53 patients, neither clinical nor pathologic features were associated with increased locoregional recurrence rates. Very young age, in particular, was not significantly associated with increased locoregional recurrences, in contrast with a previous study of unselected young patients from the Institut Curie [6].

RNA expressions of 17 target genes were studied here with RT-PCR for their association with locoregional control. They are involved in hormone signaling, either directly (*ERα, ERβ, PR, NCoA3/AIB1, NCoR, GATA3*, *CYP19/aromatase, SKP2*) or indirectly (*HER *family, *IGF1-R*) or in proliferation (*MKi67, cyclins E*, and *CAF-1*). Classic hormone receptors (*ERα, PR*) have been extensively explored in breast cancers [10]. In contrast with previously published studies [25], a high frequency of young women in this series had positive hormone receptors (*ERα *and *PR *in 79% and 81% of cases with immunohistochemistry, and in 75% and 82% of cases with RT-PCR).

Among the genes activated in response to ER activation (*NCoA3/AIB1*, *NCoR, SKP2, GATA3, CYP19/aromatase*), only the absence of CYP19/aromatase was significantly associated with an increased locoregional recurrence rate (*P *= 0.003; relative risk = 0.49; 95% CI, 0.29 to 0.82). Given the small size of this series, it also is noteworthy that the level of *GATA-3 *was associated with a trend toward an increased locoregional recurrence rate (*P *= 0.06; relative risk = 1.49; 95% CI, 0.02 to 2.39). CYP19/aromatase plays a critical role in breast cancer development by converting androgen into estrogens. *CYP19/aromatase *mRNA and protein have previously been detected in both tumor stroma and parenchymal cells in breast cancer tissue [26]. Previous data demonstrated that *CYP19/aromatase *mRNA is correlated with CYP19/aromatase enzymatic activity in cultured breast tumor fibroblasts [27]. The importance of *in situ *estrogen production has been demonstrated in breast carcinoma [28], where it is higher than that in normal breast tissue [29,30]. Inhibition of the aromatase pathway is considered to be clinically useful to reduce progression of breast tumors in postmenopausal women [31]. An apparently paradoxic finding was that a low level of *CYP19/aromatase *transcripts in this population of young, premenopausal patients was significantly associated with an increased locoregional recurrence rate. However, preclinical studies demonstrated that estrogens or estrogenic compounds repress the transcriptional control of *CYP19/aromatase *[32,33]. It can be hypothesized that, in premenopausal women, who already have high plasma estradiol levels, the level of aromatase transcripts may be inversely correlated with the plasma estradiol level [34]. Low tumor expression of *CYP19/aromatase *would reflect a high level of circulating estrogen and would therefore be associated with poor outcome. This finding is supported by a study by Zhang and colleagues [35] in a series of 162 invasive ductal breast carcinomas that showed that aromatase mRNA levels were lower in patients younger than 50 years, with tumors larger than 2 cm and with axillary lymph node involvement [35].

However, the association between tumor aromatase expression and outcome remains controversial; no consistent correlation between aromatase immunoreactivity, activity, or mRNA level and known clinicopathologic factors or outcome has been conclusively reported [35-40]. Some authors have even reported, in univariate analysis, a correlation between high tumor aromatase activity and poor outcome [37,40].

The association between tumor aromatase activity or expression and estrogen receptors is also very controversial, as some authors have reported a positive correlation [35,36,40], whereas others have reported no correlation [37], or even an inverse correlation [39]. In the present series, no correlation was observed between aromatase expression and either age, hormone receptors, or histologic grade (data not shown).

*GATA3 *is a transcription factor involved in human growth and differentiation. Gene-expression profiling has shown that *GATA3 *is highly expressed in luminal A and B subtypes of cancer and closely related to *ERα *[41-43]. This study, the first to our knowledge to use quantitative RT-PCR in a population of young patients, showed a trend toward an association between a high level of *GATA3 *expression and higher locoregional recurrence rates. The favorable outcome in very young patients with ER-positive breast cancers is a controversial issue. Aebi and associates [44] reported a series of 3,500 premenopausal women treated in four randomized trials from the International Breast Cancer Study Group (IBCSG). They found that younger patients with ER-positive tumors had a significantly poorer disease-free survival than did younger patients with ER-negative tumors.

In addition, conflicting data have been published regarding the independent prognostic value of *GATA3 *in unselected breast cancer. Some studies found *GATA3 *to be associated with good prognosis [45,46]. The study by Voduc and colleagues [47] found no independent prognostic value of *GATA3 *in 3,119 breast cancer patients with immunohistochemistry on tissue microarrays.

*ERβ *expression in breast (normal and tumor) and the relation between ERβ and other clinicopathologic features and its role in hormone therapy have been extensively investigated (recently reviewed by Zhao and others [48]). A consensus seems to have been reached regarding the protective role of *ERβ *against breast cancer development. No clinically validated cut-off has been defined for *ERβ *; transcript levels are generally lower in tumor tissue than in normal tissue. The loss of *ERβ *expression by promoter methylation, frequently observed in breast tumors, has led to the hypothesis that *ERβ *is a possible tumor-suppressor gene [49]. The *ERβ *transcript level in breast cancers was analyzed in a recent study by Anders and colleagues [50]. With clinically annotated microarray data from 200 early-stage breast cancers in women, they observed that the *ERβ *transcript level was statistically lower in young women (younger than 45 years) than in older women (older than 65 years; *P *= 0.02). As expected from these data, the present study confirmed the protective effect of *ERβ *in terms of locoregional control.

In younger women with breast cancer, a higher incidence of growth-factor receptors, namely *HER2*, was observed both in terms of protein expression, associated with a more-aggressive phenotype [51,52], and of *HER2 *gene expression with no predictive value for DFS [50].

Interestingly, the *HER *family and *IGF1R *have been shown to be intertwined with the estrogen-mediated signaling pathway [53].

Hormone receptors and growth-factor receptors act as mitogens, promoting cell proliferation in normal tissue and in breast carcinomas. Strong evidence suggests that *IGF1*, *HER*, and estrogen-mediated signaling are closely connected [54]. *HER1 *(or *EGFR*) has been described as both a prognostic marker and a predictor of hormone-therapy resistance in breast cancer [55]. However, downregulation of *EGFR *has never been explored. The present study showed a trend toward an impact of low *EGFR *expression on locoregional recurrence in young women. No other effect was observed for any of the other growth-factor receptors.

Proliferation markers, included in the definition of histologic grading [14], are also prognostic factors associated with poor outcome, high locoregional recurrence rates [5,56], and predictive factors of the response to chemotherapy [15,57]. The levels of gene expression of two subunits of *CAF-1 *(*p150 *and *p60*) [58] and other proliferation markers (the classic *KI67*, *cyclins E1 *and *E2*, and *SKP2*) were well correlated in the present study (data not shown) but were not associated with a higher risk of locoregional recurrence. One explanation could be a lack of statistical power, as most patients (68%) in this series had grade 3 tumors.

The search for a signature associated with local recurrence for premenopausal women treated with breast-conserving therapies by using high-throughput gene-expression analyses is ongoing [23,24,59]. The fact that gene-expression levels of neither *CYP19/aromatase *nor *GATA3 *were found to be associated with risk of local recurrences in the two studies [23,24] that gave information about the patients' age could reflect either the lower sensitivity of microarray data compared with RT-PCR or a difference in the studied populations. Correlations between quantitative RT-PCR measures and microarray data have been deceiving. Koscielny and associates [60] investigated the correlation between gene expressions evaluated by the qRT-PCR and microarrays in 42 colon tumors. Of 39 genes randomly selected for analysis by qRT-PCR, seven showed a correlation between microarrays and quantitative RT-PCR of 0.5 or less. The authors concluded that microarray and qRT-PCR data are not deterministically related and therefore not interchangeable.

## Conclusions

The present results highlight the role of estrogen-signaling pathways, mainly *CYP19/aromatase, GATA3*, and *ERβ*, in the risk of recurrence in young women with breast cancer. These preliminary results must be confirmed in a larger prospective study. One hypothesis would be that the higher the level of circulatory estrogen, the higher the risk of locoregional recurrence, whereas low tumor expression of aromatase and high tumor expression of *GATA3 *would reflect only the high plasma estrogen levels. However, recent data also give credit to the fact that breast cancer arising in young women is a distinct biologic entity driven by specific oncogenic pathways [50,61]. In conclusion, our results demonstrate that the hormone status is of extreme importance for the prognosis of premenopausal breast cancer patients. Although these findings must be confirmed in a larger prospective study on patients younger than 40 years, assessing the expression levels of hormone-related genes, among which aromatase, might contribute to predict disease outcome in young breast cancer patients.

## Abbreviations

*CAF-1*: *chromatin assembly factor 1*; *CCNE1*: *cyclin E1*; *CCNE1*: *cyclin E2*; CI: confidence interval; *CYP19*: *aromatase*; EORTC: European Organisation for Research and Treatment of Cancer; *ER*: *estrogen receptor*; *GATA3*: *GATA-binding protein 3*; Gy: Gray; *HER*: *human epidermal receptor*; HR: hormone receptor; *IGF1R*: *insulin-like growth factor 1-receptor*; *MKI67*: *antigen identified by monoclonal antibody ki-67*; *NCoA3/AIB1*: *nuclear receptor co-activator 3*; *NCoR*: *nuclear receptor co-repressor*; *PR*: *progesterone receptor*; Pts: patients; RR: relative risk; RT-PCR: reverse transcriptase polymerase chain reaction; *SKP2*: *S-phase kinase-associated protein 2*; *TBP*: *TATA box-binding protein*.

## Competing interests

The authors declare that they have no competing interests.

## Authors' contributions

MAB acquired clinical data and obtained funding for the study. MAB and PDC designed the study and drafted the manuscript. BSZ performed all histopathologic reviews. LDK, CTR, and CB performed all RT-PCR experiments and analyzed the data. AS and NS performed all statistical analyses. MAB, LDK, PDC, AD, NS, and AS analyzed and interpreted the data. MAB, LDK, PDC, AS, BSZ, AD, GA, PC, RS, NS, and AF helped in acquiring the data and critically revised the manuscript for important intellectual content. All authors read and approved the final manuscript.

## Supplementary Material

Additional file 1 Table listing primer and probe sequences, PCR efficiencies, regression coefficient and references. Click here for file

Additional file 2 Provides information from publicly available gene-expression studies of women, younger than 40 years old, treated with breast conserving treatments and whole breast radiotherapy. Click here for file
