# Supplementary material for: Tumor aromatase expression as a prognostic factor for local control in young breast cancer patients after breast-conserving treatment
Source: Breast Cancer Res. 2009 Jul 28;11(4):R54. doi: 10.1186/bcr2343 (PMC2750115; doi:10.1186/bcr2343)
Supplement: Additional file 1 — Table listing primer and probe sequences, PCR efficiencies, regression coefficient and references. [file bcr2343-S1.doc]

Additional data file 1: primer and probe sequences, PCR efficiencies, regression coefficient and references

|  |  |  |  | PCR efficiencies* | Regression  coefficient of  standard curves | Range of  Expression  Values  (E^∆∆CT^ x 10^-3^) | Ref |
| --- | --- | --- | --- | --- | --- | --- | --- |
| ERα | F | CCCAGCTCCTCCTCATCCT | | 98% | -3.36 |  |  |
|  | R | GGCTAGTGGGCGCATGTAG | | 97% | -3.39 | 5 to 4276 |  |
|  | Probe | AGTGCAAGAACGTGGTGCCCCTCTA | |  |  |  |  |
| Erβ | F | AAGAATATCTCTGTGTCAAGGCCATG | | >100% | -3.28 |  | [62] [19] |
|  | R | GGCAATCACCCAAACCAAAG | | >100% | -3.27 | 45 to 1495 |  |
|  | Probe | TTGCTGAACGCCGTGACCGATG | |  |  |  |  |
| PR | F | GAACCAGATGTGATCTATGCAGGA | | 100% | -3.33 |  | [62] [19] |
|  | R | CGAAAACCTGGCAATGATTTAGAC | | 95% | -3.44 | 0 to 712 |  |
|  | Probe | ACCTGACACCTCCAGTTCTTTGCTGACAAG | |  |  |  |  |
| HER1 | F | TCCCCGTAATTATGTGGTGACAGATC | | 89% | -3.62 |  |  |
|  | R | ACCCCTAAATGCCACCGGC | | 91% | -3.56 | 24 to 1858 |  |
|  | Probe | CAGCTATGAGATGGAGGAAGACGGCGT | |  |  |  |  |
| HER2 | F | CAACCAAGTGAGGCAGGTCC | | 95% | -3.45 |  | [63] |
|  | R | CTGGATCAAGACCCCTCCTTT | | 96% | -3.41 | 379 to 65440 |  |
|  | Probe | AGAGGCTGCGGATTGTGCGA | |  |  |  |  |
| HER3 | F | ATGAGGGCGAACGACGC | | 94% | -3.47 |  |  |
|  | R | TACAGTGTCTGGTATTGGTTCTCAGC | | 94% | -3.48 | 96 to 1882 |  |
|  | Probe | TGAATGGCCTGAGTGTGACCGGC | |  |  |  |  |
| HER4 | F | CCCATGGCTGCCTGTTG | | 98% | -3.38 |  |  |
|  | R | CTAGCCCAAAATCTGTGATTTTCAC | | 98% | -3.36 | 4 to 11539 |  |
|  | Probe | TCATCGGGATTTGGCAGCCCGTA | |  |  |  |  |
| Ki67 | F | ATTGAACCTGCGGAAGAGCTGA | | >100% | -3.29 |  |  |
|  | R | GGAGCGCAGGGATATTCCCTTA | | 97% | -3.4 | 65 to 5783 |  |
|  | Probe | ACGACATGAAAACCAACAAAGA | |  |  |  |  |
| Cyclin E1 | F | TGCGAGCAATTCTTCTGGATT | | 97% | -3.34 |  |  |
|  | R | GTGTCGCCATATACCGGTCA | | 100% | -3.317 | 41 to 4610 |  |
|  | Probe | TTCACAGGGAGACCTTTTACTTGGCACA | |  |  |  |  |
| Cyclin E2 | F | GCAGCAGCAGTCAGTATTCTGTACT | | 98% | -3.37 |  |  |
|  | R | TCAAATAGCTCAGCTTTTAGATCTGTGT | | 100% | -3.33 | 341 to 9837 |  |
|  | Probe | CTCTAATGAATCAATGGCTAGAA | |  |  |  |  |
| AIB1 | F | AGCAAGGCAATTTTCCTCTCC | | 98% | -3.37 |  | [63] |
|  | R | TCAAGTGCCTGTCGGCTCT | | 97% | -3.39 | 911 to 7310 |  |
|  | Probe | ACCCCGGACAAACACCCCCAA | |  |  |  |  |
| Ncor | F | AGAGAGCAGCCACTGGGTCT | | 96% | -3.43 |  |  |
|  | R | AGTTGTACGGCCTGGGAGG | | 97% | -3.4 | 527 to 5522 |  |
|  | Probe | CAGCAACGAGAGGAATCATTGACCTGA | |  |  |  |  |
| CYP19 | F | TGGAAAACAACTCGACCCTTCT | | >100% | -3.141 |  | [19] |
|  | R | CACAGACTGTGACCATACGAACAA | | >100% | -3.29 | 7 to 320 |  |
|  | Probe | TGAAAGCTCTGTCAGGCCCCGG | |  |  |  |  |
| SKP2 | F | GAGTCCCATGAAACACCTGGAA | | >100% | -3.203 |  |  |
|  | R | CTGAAGAGCAAAGGGAGTGACA | | 100% | -3.325 | 501 to 6036 |  |
|  | Probe | CTCTCGATTTAGCTTAGGCCTGCGGACA | |  |  |  |  |
| CAF-1 p150 | F | CAAAGCAGCACAGCAGTACCA | | 94% | -3.47 |  |  |
|  | R | GATCTCTTTGCAGTCTGAGCTTGTT | | 98% | -3.367 | 296 to 2531 |  |
|  | Probe | TCCACGCCCCTCCGCAGAATAACTA | |  |  |  |  |
| CAF-1 p60*** | F | CGGACACTCCACCAAGTTCT | | 98% | -3.36 | 852 to 10056 | [58] |
|  | R | CCAGGCGTCTCTGACTGAAT | |  |  |  |  |
| IGF1R | HS00609566_m1 | | Applied Biosystems | 96% | -3.43 |  |  |
|  |  |  |  | 91% | -3.56 | 272 to 44473 |  |
| GATA3 | HS00231122_m1 | | Applied Biosystems | 99% | -3.38 |  |  |
|  |  |  |  | 96% | -3.41 | 6 to 1430 |  |
| TBP | F | CACGAACCACGGCACTGATT | |  |  |  | [19] |
|  | R | TTTTCTTGCTGCCAGTCTGGAC | | 96% to 100 | -3,41 to -3,33 |  |  |
|  | Probe | TGTGCACAGGAGCCAAGAGTGAAGA | |  |  |  |  |

*All probes are labelled with FAM/TAMRA with two exceptions(KI67 and cyclin E2) that are MGB probes*

* Two different experiments. ** Eight different experiments. ***CAF-1 p60 has been analyzed separately using the SYBRGreen method.

The comparison of duplicates for all gene expression shows that the CV are always < 2.5%.

The interassay CV in these experimental conditions is <5%.
