# Supplementary material for: Tumor aromatase expression as a prognostic factor for local control in young breast cancer patients after breast-conserving treatment
Source: Breast Cancer Res. 2009 Jul 28;11(4):R54. doi: 10.1186/bcr2343 (PMC2750115; doi:10.1186/bcr2343)
Supplement: Additional file 2 — Provides information from publicly available gene-expression studies of women, younger than 40 years old, treated with breast conserving treatments and whole breast radiotherapy. [file bcr2343-S2.doc]

Additional data file 2: Information from publicly available gene-expression studies of women, younger than 40 years old, treated with breast conserving treatments and whole breast radiotherapy (Kreike et al. 1A and 1B [23] and Nuyten et al. 2A and 2B [24]). Box-plots of gene-expression level for both CYP19/Aromatase (1A and 2A) and GATA3 (1B, 2B) in patients with (red) or without (blue) breast cancer local recurrences.

**1A**

**1B**

**2A**

**2B**
